# Completely Diverted Tube Ileostomy Versus Conventional Loop Ileostomy

**DOI:** 10.7759/cureus.30997

**Published:** 2022-11-02

**Authors:** Alisina Bulut, Wafi Attaallah

**Affiliations:** 1 General Surgery, Bogazici Academy for Clinical Sciences, Istanbul, TUR; 2 General and Colorectal Surgery, Marmara University School of Medicine, Istanbul, TUR

**Keywords:** rectal cancer, anastomotic leakage, fecal diversion, loop ileostomy, tube ileostomy

## Abstract

Purpose

Diverting ileostomies are commonly performed to prevent morbidity and mortality caused by colorectal anastomotic leakage. However, many complications may develop due to loop ileostomy itself and its reversal. In this study, we aimed to compare the outcomes of completely diverted tube ileostomy and conventional loop ileostomy.

Methods

The study was designed prospectively, and operations were performed by the same surgeon at a single center. Completely diverted tube ileostomy with the rubber strip was performed in 20 consecutive patients, and loop ileostomy was performed in the next 20 consecutive patients who needed diverting stoma. The primary outcome of the study is to compare the overall complication rates in both techniques. Length of hospital stay, achieving complete diversion, and length of time with a stoma were evaluated as secondary outcomes.

Results

There were no significant differences in the demographic characteristics between the two groups. Complete diversion was achieved in both groups. The number of patients who developed any kind of complications during the observation period was significantly higher in the loop ileostomy group in comparison with the tube ileostomy group (13 (65%) versus 3 (15%), respectively (p=0.002)). The median time with a stoma was significantly higher in the loop ileostomy group compared to the tube ileostomy group (270 days (range: 56-443) versus 21 days (range: 14-28), respectively (p<0.001)).

Conclusion

Completely diverted tube ileostomy causes fewer complications, provides a cost advantage, and does not require surgery for stoma closure.

## Introduction

The incidence of colorectal anastomotic leakage reported in the literature ranges between 1% and 25% [1]. Anastomotic leakage following colorectal anastomosis is associated with serious morbidity and mortality and may result in a permanent stoma [2]. Although loop ileostomy does not prevent a leak, it is a commonly used procedure to prevent serious complications of anastomotic leakages, such as fecal peritonitis and septicemia [3-5]. However, loop ileostomy is associated with many complications such as poor stoma siting, dehydration, electrolyte abnormalities, skin irritation, retraction, ischemia, stoma stenosis, parastomal hernia, prolapse, and psychological detriment [6]. Moreover, there is a need for a second operation to reverse the stoma, which is associated with significant morbidity and mortality [7,8]. Alternative techniques for fecal diversion were described to avoid complications associated with a loop ileostomy. One of these alternatives is tube ileostomy. However, many of the described tube ileostomy techniques were not sufficient to provide complete fecal diversion. In a previous study, we described a new technique of completely diverted tube ileostomy for the protection of colorectal anastomosis [9]. The aim of this study is to compare outcomes of conventional loop ileostomy with recently described completely diverting tube ileostomy.

This article was previously presented as a meeting abstract at the 2021 Turkish Colon and Rectum Surgery Annual Congress on November 30 to December 4, 2021. Also, this article was previously posted to the Research Square preprint server on June 1, 2022.

## Materials and methods

Study design

**Figure 1:**
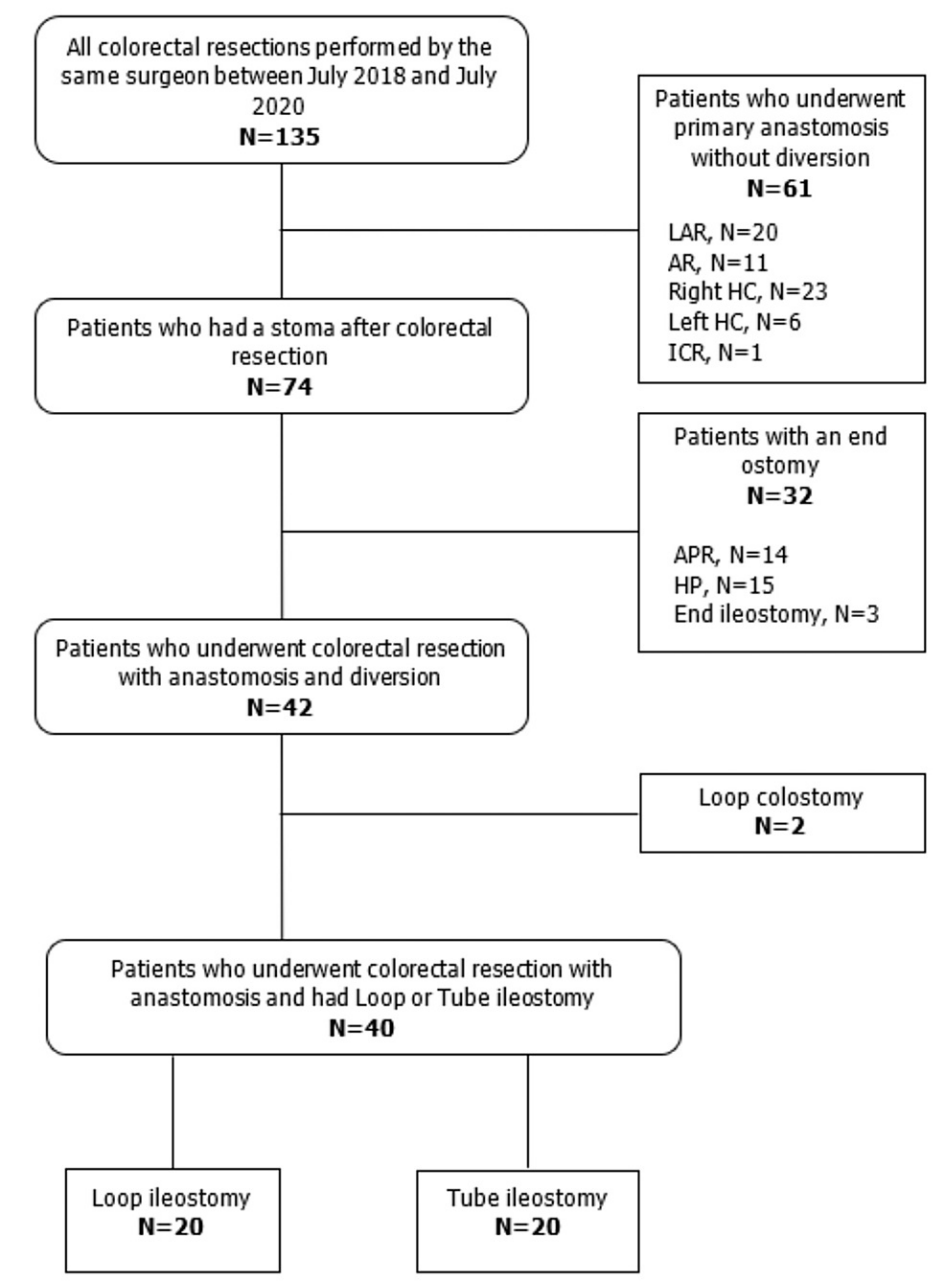
Study selection flowchart LAR, low anterior resection; AR, anterior resection; HC, hemicolectomy; ICR, ileocecal resection; APR, abdominal perineal resection; HP, Hartmann’s procedure

This prospective study was conducted in the General Surgery Clinic at Marmara University Education and Research Hospital in Turkey. Ethics committee approval was obtained from Marmara University Clinical Research Ethics Committee (approval number: 09.2020.663). The study included a total of 40 patients who underwent diversion using either tube ileostomy or loop ileostomy following colorectal resection from July 2018 to July 2020. To prevent selection bias, two groups of consecutive patients were formed. Twenty consecutive patients were included in each group of tube and loop ileostomy (Figure 1).

**Figure 2:**
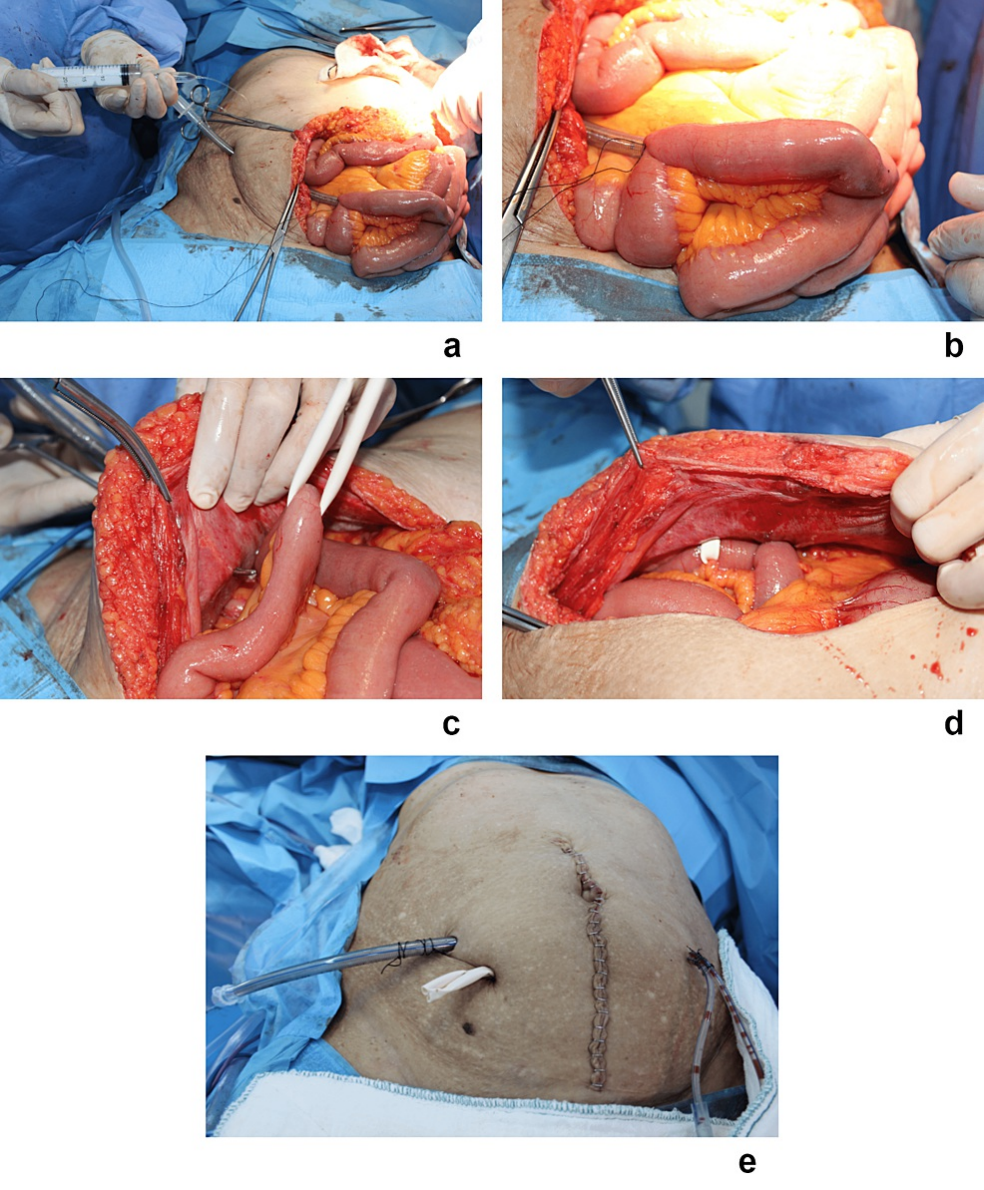
Intraoperative stages of tube ileostomy (a) Inflating the balloon by placing the spiral endotracheal tube toward the ileum in the proximal direction. (b) Tension in the ileum wall after the balloon is inflated. (c) Suspending the ileum with a rubber strip distal to the tube. (d) View of the rubber strip fixed to the abdominal wall. (e) Completed image of completely diverted tube ileostomy with rubber strip.

The tube Ileostomy technique was described and illustrated in detail in the previous study (Figure 2) [9]. It can be briefly described as after resection and anastomosis were completed, a reinforced (spiral) endotracheal cuffed tube (Fuzhou Kanglite Surgical Plastic Cement Co., Ltd., Fuzhou, China) with an inner diameter of 7.5 mm was inserted into the ileum at a point 20 cm proximal to the ileocecal valve, with the tube tip directed proximally. The tube was secured to the bowel wall using a purse string, and the bowel was then fixed to the parietal peritoneum with two stitches. The balloon was inflated with saline to fill up the lumen of the ileum (Figure 2a). While intestinal occlusion was provided by inflating the balloon, care was taken to ensure that the ileum wall was not in tension to cause ischemia (Figure 2b). The ileal lumen was occluded with a rubber strip (Penrose drainage tube, size 1/4, TRAF Company, Gujarat, India) that passed through the mesentery (Figure 2c) and was brought out of the peritoneal cavity through a stab incision on the abdominal wall. The taut strip was then fixed to the abdominal wall (Figure 2d). Finally, the tube was secured to the skin (Figure 2e) and connected to a nylon bag. Loop Ileostomy was performed using the standard technique commonly used in the practice [10].

Patient evaluation

All patients underwent colorectal resection with primary anastomosis. Neoadjuvant chemoradiotherapy (CRT), distal rectal tumor, comorbidities, and positive air leak test were determined as criteria for diversion. Tube ileostomy was performed in 20 consecutive patients, and loop ileostomy was performed in the following 20 consecutive patients who met these criteria.

Data related to patient age, sex, diagnosis, indication for ileostomy, comorbidity, body mass index (BMI), American Society of Anesthesiologists (ASA) score, tumor location, neoadjuvant CRT, surgical approach (open/laparoscopic), and postoperative ileus were collected prospectively in both groups. Additionally, stoma-related complications (i.e., prolapsus, stricture, retraction, parastomal hernia, mucocutaneous separation, and skin maceration) and the timing of stoma closure were assessed in the loop ileostomy group. Stoma closure complications were described as the need for laparotomy during the reversal surgery, surgical site infection, visceral injury during reversal surgery, and postoperative ileus. In the tube ileostomy group, the timing of the rubber strip removal, tube removal, and spontaneous fistula closure was recorded. Rectoscopy was performed to confirm anastomotic healing before tube removal. Stoma-related complications in the tube ileostomy group were determined as skin maceration, peristomal infection, and peritonitis. Complete diversion was defined as no defecation in the presence of diversion.

We compared the severity of complications between the two groups using the Clavien-Dindo classification.

To evaluate the psychosocial adjustment of individuals living with a stoma, we used the Ostomy Adjustment Inventory-23 (OAI-23) that was developed by Simmons et al. [11]. This scale is composed of 23 items, with higher scores indicating better adjustment of the patients to the stoma. Because the items of the scale are not suitable for tube ileostomy patients, we used the scale only in the loop ileostomy group.

Statistical analysis

Data were analyzed using Statistical Package for the Social Sciences (SPSS) for Windows version 23 (IBM SPSS Statistics, Armonk, NY, USA). All statistical tests were two-sided, and values of p<0.05 were considered to be signiﬁcant. Mann-Whitney U test or t-test was used for continuous data, and the Fisher exact test or chi-squared test was used for categorical data.

Outcomes

The primary outcome of this study was to compare the complication rates between tube ileostomy and loop ileostomy. The secondary outcomes were to compare the rate of stoma-free time, duration of hospital stay, and stoma output. The ability to provide complete diversion was evaluated in both groups.

## Results

In total, 135 colorectal resections were performed by the same surgeon at Marmara University Education and Research Hospital between July 2018 and July 2020. Among those, a total of 40 patients needed a protective stoma. Tube ileostomy was performed in 20 consecutive patients, and loop ileostomy was performed in the following 20 consecutive patients. No significant differences were observed between the groups regarding age, sex, BMI, diagnosis, neoadjuvant CRT, and tumor location (Table 1).

**Table 1:** Demographic and clinical comparison of the loop ileostomy and tube ileostomy patient groups BMI, body mass index; ASA, American Society of Anesthesiologists; AV, anal verge; CRT, chemoradiotherapy

	Loop ileostomy (N=20)	Tube ileostomy (N=20)	p value
Age (year), mean	54 (range: 30-72)	58 (range: 36-76)	1
Sex			
Male	13 (65%)	14 (70%)	0.7
Female	7 (35%)	6 (30%)	
BMI (kg/m^2^), mean	27 (range: 22-35)	27 (range: 21-40)	1
ASA score			
1	5 (25%)	0 (0%)	0.05
2	15 (75%)	14 (70%)	0.7
3	0 (0%)	6 (30%)	0.02
Tumor distance from AV (cm), mean	7 (range: 2-12)	8 (range: 3-40)	1
Diagnosis			
Rectal cancer	20 (100%)	17 (85%)	1
Sigmoid cancer	0 (0%)	3 (15%)	
Preoperative CRT			
Yes	11 (55%)	9 (45%)	0.5
No	9 (45%)	11 (55%)	
Type of approach			
Open	19 (95%)	19 (95%)	1
Laparoscopic	1 (5%)	1 (5%)	

The vast majority (93%) of the whole cohort was diagnosed with rectal cancer. Complete diversion was achieved in 19 (95%) patients in the loop ileostomy group and 20 (100%) patients in the tube ileostomy group, and there was no significant difference. Postoperative ileus developed in two patients in each group and resolved spontaneously. The median stoma output (which was recorded for 14 days postoperatively) was significantly higher in the loop ileostomy group than in the tube ileostomy group (775 mL/day (range: 450-1,500) and 588 mL/day (range: 150-1,650), respectively (p=0.02)) (Table 2). No significant difference was found between the two groups regarding the total length of hospital stay (p=1.0).

**Table 2:** Comparison of postoperative outcomes

	Loop ileostomy (N=20)	Tube ileostomy (N=20)	p value
Complete diversion			
Yes	19 (95%)	20 (100%)	1
No	1 (5%)	0 (0%)	
Daily output (mL), mean	775 (range: 450-1,500)	588 (range: 150-1,650)	0.02
Time to stoma reversal (day), mean	270 (range: 56-443)	21 (range: 14-28)	<0.001
Length of hospital stay (day), mean	12 (range: 5-33)	11 (range: 5-30)	1
Follow-up period (month), mean	11 (range: 7-16)	24 (range: 10-28)	<0.001

The median time to stoma reversal was significantly higher in the loop ileostomy group than in the tube ileostomy group (270 days (range: 56-443) and 21 days (range: 14-28), respectively (p<0.001)).

During a median follow-up of 11 months, the stoma was closed in eight (40%) patients, while the remaining 11 (55%) patients are still living with a stoma in the loop ileostomy group mostly due to ongoing chemotherapy. One patient died in the loop ileostomy group due to candida sepsis while living with a stoma. In the tube ileostomy group, the tubes of all patients were removed, and stoma-free life was provided.

The total number of patients with any complications regarding stoma was significantly higher in the loop ileostomy group compared to the tube ileostomy group (13 (65%) and 2 (10%), respectively (p<0.001)) (Table 3). According to the Clavien-Dindo classification, 10 (50%) patients had grade I complications (including skin maceration, dehydration, and prolapse that did not require intervention) in the loop ileostomy group, whereas there were none in the tube ileostomy group (p<0.001).

**Table 3:** Comparison of complications between both groups

	Loop ileostomy (N=20)	Tube ileostomy (N=20)	p value
Number of patients with stoma-related postoperative complications	13 (65%)	2 (10%)	<0.001
Stoma-related complications graded according to the Clavien-Dindo classification			
Grade 1	10 (50%)	0 (0%)	<0.001
Grade 2	1 (5%)	2 (10%)	1
Grade 3a	0 (0%)	0 (0%)	<0.05
Grade 3b	3 (15%)	0 (0%)	
Grade 4a	2 (10%)	0 (0%)	
Grade 4b	0 (0%)	0 (0%)	
Grade 5	0 (0%)	0 (0%)	
Skin maceration	9 (45%)	2 (10%)	0.013
Postoperative ileus	2 (10%)	2 (10%)	1

**Figure 3:**
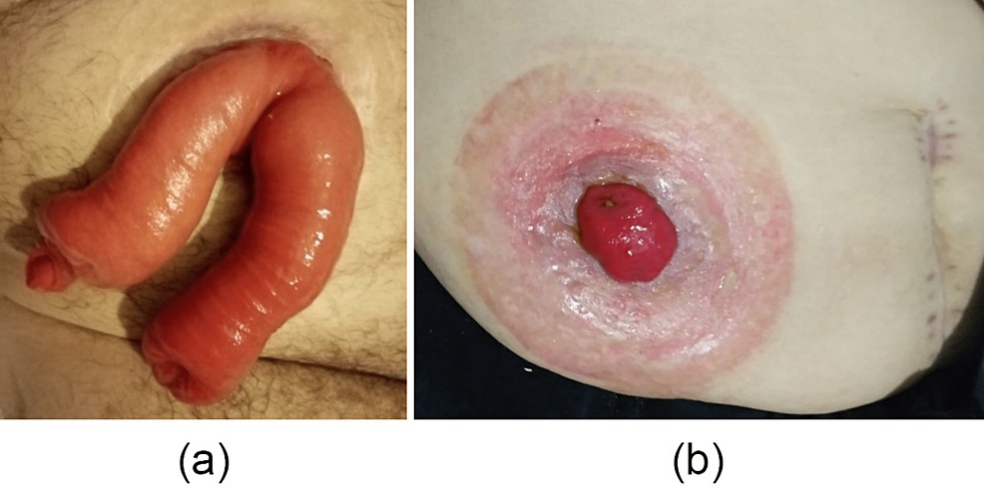
Loop ileostomy complications: (a) prolapsus and (b) skin maceration

Among patients with loop ileostomy, stoma prolapse was observed in two (10%) patients, skin maceration in nine (45%), parastomal hernia in two (10%), and stoma reversal complications in three (15%) patients (Figure 3).

However, only two (10%) patients with tube ileostomy had complications after surgery, which were a peristomal infection (Clavien-Dindo grade II) that healed with antibiotherapy. Among patients with loop ileostomy, Clavien-Dindo grade IIIb complications were observed in three (15%) patients (prolapse in one (5%) patient and incarcerated parastomal hernia in two (10%) patients; all required surgical intervention), and grade IVa complications were observed in two (10%) patients (organ failure and sepsis following stoma reversal surgery as a result of anastomotic leakage). There were no grade III-IV complications in the tube ileostomy group.

Because skin maceration is a complication that could arise in both groups, it was evaluated between the two groups as a separate entity. Skin maceration was observed in nine (45%) patients of the loop ileostomy group and two (10%) patients of the tube ileostomy group, which showed a significant difference in favor of the tube ileostomy group (p=0.013).

The median score of OAI-23 among patients with loop ileostomy was 43 (range: 13-69), and the score was lower than 50 in 84% of the patients. Most of the patients were unable to adjust to the stoma.

The minimal monthly cost of an ostomy kit and paste for one patient at the date of the study was about $35. During the follow-up period, the median cost of an ostomy kit and paste for 20 loop ileostomy patients was about $1,000 per patient. Furthermore, 11 (55%) patients who still have a stoma continue to bear this cost. Meanwhile, the median cost of an endotracheal tube and nylon bag for tube ileostomy is $10.

## Discussion

This prospective study compared two groups of a total of 40 patients undergoing diversion using either tube or loop ileostomy following colorectal resection. It was concluded that tube ileostomy with rubber strip provides fecal diversion as effectively as loop ileostomy but may cause fewer complications.

To the best of our knowledge, this is the first study to compare loop ileostomy with completely diverting tube ileostomy achieved through temporary occlusion of the distal ileum using a flexible rubber strip.

The prospective design, having two comparative groups, and minimizing selection bias by including consecutive patients were also other strengths of this study. The limitations of this study were the small sample size and short follow-up period. Since there is no standardized adjustment scale for tube ileostomy, stoma-related psychosocial problems could not be evaluated in these patients. We can also consider this situation as a limitation of our study.

Tube ileostomy was uncommonly reported in the literature, and no standard technique was described. Using an endotracheal tube was previously reported in some studies, and it was supposed that the inflated balloon of the tube could occlude the intestinal lumen [12-15]. However, the inflated balloon could not effectively occlude the lumen for a long time, particularly as the return of motility to the ileum results in the propulsion of its contents past the balloon [16]. Zhou et al. [17] presented a technique to overcome this problem. They used a single line of staples along with tube ileostomy to prevent the early flow of feces to the anastomotic region. They suggested that after the recovery of intestinal motility, feces will enter the distal limb and will gradually disrupt the staple line; thus, recanalization will occur spontaneously.

Although recanalization of the staple line was observed in all patients, the authors stated that the fate of the staples was unclear. Another weakness of this technique is that the operating surgeon cannot decide the duration of the diversion. There is a possibility that recanalization may not occur at all or it may occur too early, resulting in ineffective diversion. For these reasons, we used an easily removable rubber strip to occlude the ileum instead of a single row of staples, in the technique that we demonstrated in our previous pilot study, and complete diversion was achieved in all patients. Rubber band removal time was determined based on the healing of the anastomosis line of each patient (anastomosis healing was decided by controlling with rectoscopy). Therefore, effective diversion can be achieved in the desired time.

In previous studies, different materials such as gastrostomy tubes, Foley catheters, and endotracheal tubes were used in tube ileostomy. The internal diameters of the tubes range between 18F (6 mm) and 28F (9.3 mm) [5]. These different tube types could not overcome the problem of kinking and the associated obstruction. Hua et al. [18] discussed that soft and thin tubes could be easily obstructed and did not provide function. In our study, a reinforced (spiral) endotracheal cuffed tube (Fuzhou Kanglite Surgical Plastic Cement Co., Ltd., Fuzhou, China) with an inner diameter of 7.5 mm was used. Owing to its flexible spiral structure, kinking and obstruction of the tube were prevented. In addition, a spiral tube allows patients to bend the tube and hide it under their clothing, which improves patient comfort.

Previous studies reported that tube ileostomy could not prevent the development of fecal peritonitis caused by anastomotic leakage, and as a result, loop ileostomy has been preferred over tube ileostomy [12-14,19]. In this study, fecal peritonitis did not occur in any patient.

Although a diverting loop ileostomy is intended to be temporary, large series studies reported that only two-thirds of all temporary stomas were closed, whereas more than 30% of all patients kept their stomas permanently or died before closure [20]. In this study with relatively short follow-up, we observed that more than half of the patients continue to live with loop ileostomy. In three (15%) patients, loop ileostomy became permanent due to disease progression, which is consistent with the literature [20]. In the tube ileostomy group, the tube could be removed in a median of 21 days, and all patients became stoma-free. These results suggest that tube ileostomy can reduce the risk of a permanent stoma.

There is plenty of data in the literature on the complications of loop ileostomy [21-23]. In the systemic review by Malik et al. [23], 18 randomized controlled studies were assessed, and stoma-related complications by stoma types were presented. Complication rates were reported as follows: peristomal skin complication, 14% (5.6%-37.8%); high-output stoma, 2.4% (0%-18.5%); parastomal hernia, 2.4% (0%-13.3%); stoma retraction, 3.1% (0%-10.8%); and stoma prolapse, 0% (0%-5.4%). The study showed that complications have a negative impact on the patient and healthcare professionals and can lead to cost disadvantages.

In a retrospective study by Rondelli et al. [15] comparing loop ileostomy to tube ileostomy using a Foley catheter, the two methods were evaluated in terms of complications. When stoma-related complications were evaluated separately, the parastomal hernia was found in 12 (17%), intestinal obstruction in one (0.01%), and stoma stenosis in two (0.02%) patients of the loop ileostomy group. Although they stated that stoma-related complications such as parastomal hernia were significantly higher in the loop ileostomy group (loop and tube: 12 and 0 (p<0.02)), their study lacks information on skin infection or skin maceration. In our study, stomal prolapse was observed in two (10%), parastomal hernia in two (10%), and skin maceration in nine (45%) patients of the loop ileostomy group.

The most likely reason for the higher skin maceration rate in comparison with the literature may be due to the low recognition rate of maceration by health professionals and physicians. In contrast, the incidence of parastomal hernia and prolapse is consistent with the literature [23]. Both tube ileostomy and loop ileostomy have specific complications. For instance, prolapsus is not an expected complication of tube ileostomy. Therefore, it is not reasonable to get significant results by directly comparing these method-specific complications. To overcome this problem, we used the Clavien-Dindo classification to assess the severity of different complications. To the best of our knowledge, this study is the first one to use the Clavien-Dindo classification to compare complications that are associated with tube ileostomy and loop ileostomy procedures.

In addition to the complications related to loop ileostomy, it also has a negative impact on the quality of life. Studies comparing loop ileostomy to tube ileostomy lack data regarding patients’ adjustment to life with a stoma. We assessed the adjustment of loop ileostomy among the patients using the OAI-23 questionnaire. Results showed that most of the patients in the loop ileostomy group had problems with adjustment to life with a stoma, encountered restrictions in social activities, and had a constant worry about the stoma.

This study showed that there is a 10-fold cost advantage between the two methods in favor of tube ileostomy. It is indisputable that if stoma reversal surgery charges are added to the cost, the difference will be even greater. This apparent cost advantage shown in this study limited to 20 patients could be of great value in further studies with a large number of patients.

In this study, it was shown that tube ileostomy provides complete fecal diversion and causes fewer stoma-related complications. However, the sample size and design of our study are not enough to state that one method is superior to the other.

## Conclusions

Completely diverted tube ileostomy causes fewer complications and provides a cost advantage compared to loop ileostomy, and surgery is not required for tube ileostomy reversal. However, randomized controlled trials with larger samples are needed to demonstrate that tube ileostomy with rubber strip is superior to loop ileostomy.

## References

[1] Paun BC, Cassie S, MacLean AR, Dixon E, Buie WD (2010). Postoperative complications following surgery for rectal cancer. Ann Surg.

[2] Perez RO, Habr-Gama A, Seid VE (2006). Loop ileostomy morbidity: timing of closure matters. Dis Colon Rectum.

[3] Gastinger I, Marusch F, Steinert R, Wolff S, Koeckerling F, Lippert H (2005). Protective defunctioning stoma in low anterior resection for rectal carcinoma. Br J Surg.

[4] Matthiessen P, Hallböök O, Rutegård J, Simert G, Sjödahl R (2007). Defunctioning stoma reduces symptomatic anastomotic leakage after low anterior resection of the rectum for cancer: a randomized multicenter trial. Ann Surg.

[5] Nachiappan S, Datta U, Askari A, Faiz O (2015). Tube ileostomy for faecal diversion in elective distal colorectal anastomosis: a systematic review and pooled analysis. Colorectal Dis.

[6] Delrio P, Conzo G (2008). Complications of ileostomy. Seminars in colon and rectal surgery.

[7] D'Haeninck A, Wolthuis AM, Penninckx F, D'Hondt M, D'Hoore A (2011). Morbidity after closure of a defunctioning loop ileostomy. Acta Chir Belg.

[8] Sharma A, Deeb AP, Rickles AS, Iannuzzi JC, Monson JR, Fleming FJ (2013). Closure of defunctioning loop ileostomy is associated with considerable morbidity. Colorectal Dis.

[9] Attaallah W, Bulut A, Uprak TK, Yegen C (2020). A new technique of completely diverted tube ileostomy for the protection of colorectal anastomosis: a pilot study. Colorectal Dis.

[10] Brunicardi FC, Andersen DK, Billiar TR (2019). Schwartz's principles of surgery 11th edition.

[11] Simmons KL, Smith JA, Maekawa A (2009). Development and psychometric evaluation of the Ostomy Adjustment Inventory-23. J Wound Ostomy Continence Nurs.

[12] Chowdri NA, Wani MA, Parray FQ, Mir SH, Wani RA (2010). Tube ileostomy as an alternative to conventional ileostomy for fecal diversion. World J Colorectal Surg.

[13] Monzón-Abad A, Gracia-Roche C, Martínez-Germán A, Barranco-Domínguez I, Sánchez-Fuentes N (2014). A preliminary study of transcaecal ileostomy as an alternative to defunctioning ostomies. Colorectal Dis.

[14] Rondelli F, Mariani L, Boni M, Federici MT, Cappotto FP, Mariani E (2010). Preliminary report of a new technique for temporary faecal diversion after extraperitoneal colorectal anastomosis. Colorectal Dis.

[15] Rondelli F, Balzarotti R, Bugiantella W, Mariani L, Pugliese R, Mariani E (2012). Temporary percutaneous ileostomy versus conventional loop ileostomy in mechanical extraperitoneal colorectal anastomosis: a retrospective study. Eur J Surg Oncol.

[16] Rangabashyam N, Gnanaprakasam D, Damodaram S (1983). Temporary defunctioning transcaecal tube ileostomy for left colon resections. Indian J Gastroenterol.

[17] Zhou X, Lin C, Chen W, Lin J, Xu J (2014). Completely diverted tube ileostomy compared with loop ileostomy for protection of low colorectal anastomosis: a pilot study. Colorectal Dis.

[18] Hua H, Xu J, Chen W, Zhou X, Wang J, Sheng Q, Lin J (2014). Defunctioning cannula ileostomy after lower anterior resection of rectal cancer. Dis Colon Rectum.

[19] Ansari MM, Ahmad S, Hasan SH, Haleem S (2011). Feasibility and outcome of proximal catheter ileostomy - a pilot study. Saudi J Gastroenterol.

[20] Kairaluoma M, Rissanen H, Kultti V, Mecklin JP, Kellokumpu I (2002). Outcome of temporary stomas. A prospective study of temporary intestinal stomas constructed between 1989 and 1996. Dig Surg.

[21] García-Botello SA, García-Armengol J, García-Granero E, Espí A, Juan C, López-Mozos F, Lledó S (2004). A prospective audit of the complications of loop ileostomy construction and takedown. Dig Surg.

[22] Kaidar-Person O, Person B, Wexner SD (2005). Complications of construction and closure of temporary loop ileostomy. J Am Coll Surg.

[23] Malik T, Lee MJ, Harikrishnan AB (2018). The incidence of stoma related morbidity - a systematic review of randomised controlled trials. Ann R Coll Surg Engl.

